# Supplementary material for: Delocalization of the Unpaired Electron in the Quercetin Radical: Comparison of Experimental ESR Data with DFT Calculations
Source: Int J Mol Sci. 2020 Mar 16;21(6):2033. doi: 10.3390/ijms21062033 (PMC7139295; doi:10.3390/ijms21062033)
Supplement: Supplementary file 1 [file ijms-21-02033-s001.pdf]

Supplementary Materials

Hydroquinone radical

xyz -1 2

1 O	2.71320000	0.00000000	0.00000000	23.695771	
2 O	-2.71320000	0.00000000	0.00000000	23.695771	
3 C	-0.68200000	-1.22670000	0.00000000	6.714908	2
4 C	0.68200000	-1.22670000	0.00000000	6.714908	3
5 C	-1.44160000	0.00000000	0.00010000	12.639612	
6 C	1.44160000	0.00000000	-0.00020000	12.639549	
7 C	-0.68200000	1.22670000	0.00000000	6.714619	5
8 C	0.68200000	1.22670000	0.00000000	6.714905	6
9 H	1.23950000	-2.15810000	0.00000000	-0.117527	
10 H	-1.23950000	2.15810000	0.00010000	-0.117509	
11 H	-1.23950000	-2.15810000	0.00000000	-0.117546	
12 H	1.23950000	2.15810000	0.00010000	-0.117497	

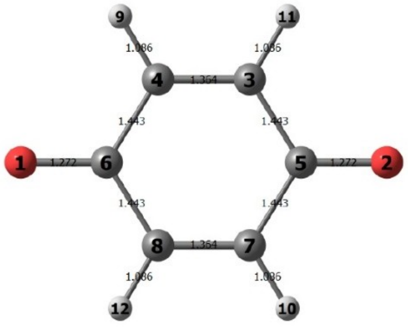

Catechol radical

xyz -1 2

1 O	1.74750000	1.37080000	0.00010000	22.934513	
2 O	1.74760000	-1.37080000	0.00000000	22.930758	
3 C	-1.79820000	0.71250000	0.00000000	10.962429	4
4 C	-0.62140000	1.41160000	0.00000000	2.692739	3
5 C	-1.79820000	-0.71250000	0.00000000	10.957973	5
6 C	0.64910000	0.74370000	-0.00030000	13.275079	
7 C	-0.62140000	-1.41170000	0.00000000	2.696376	6
8 C	0.64920000	-0.74370000	0.00010000	13.274418	
9 H	-0.61310000	2.49680000	0.00000000	-0.120978	
10 H	-2.74440000	-1.24190000	0.00000000	-0.016969	
11 H	-0.61310000	-2.49680000	0.00000000	-0.120960	
12 H	-2.74440000	1.24180000	0.00010000	-0.016809	

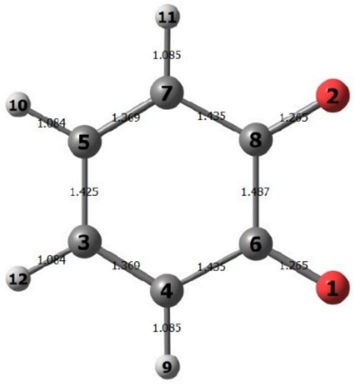

kampferol radical

xyz -3 2

1 O	-0.08480000	-0.73620000	0.00000000	3.342085	
2 O	0.78610000	2.72290000	-0.00020000	23.952931	
3 O	-4.32360000	1.57980000	0.00010000	1.581130	
4 O	-1.84470000	2.93660000	0.00000000	0.614638	
5 O	-4.08890000	-3.13070000	0.00010000	1.487120	
6 O	6.12320000	-1.00430000	0.00010000	9.052400	
7 C	-2.14480000	0.56600000	-0.00010000	0.224810	
8 C	-1.45810000	-0.67810000	-0.00010000	-1.484219	
9 C	0.71500000	0.36360000	0.00000000	19.642921	
10 C	2.10430000	0.04940000	0.00000000	1.015589	
11 C	0.12070000	1.66000000	0.00000000	12.337583	
12 C	-1.39390000	1.77580000	-0.00010000	0.201918	
13 C	-3.61970000	0.51630000	-0.00010000	0.888165	
14 C	-2.07280000	-1.89940000	0.00000000	5.839743	8
15 C	-3.51340000	-1.98780000	0.00000000	0.855905	
16 C	2.52360000	-1.31480000	-0.00040000	4.250738	2’
17 C	-4.22190000	-0.76310000	0.00000000	-0.566739	
18 C	3.13550000	1.03420000	0.00040000	4.118287	6’
19 C	3.84420000	-1.66740000	-0.00040000	1.377024	3’
20 C	4.45890000	0.68610000	0.00050000	1.017908	5’
21 C	4.89890000	-0.68490000	0.00010000	5.958370	
22 H	-1.47880000	-2.80580000	0.00000000	-0.040358	
23 H	1.77200000	-2.09410000	-0.00080000	-0.050083	
24 H	-5.30760000	-0.79940000	0.00010000	-0.011811	
25 H	2.85880000	2.07760000	0.00080000	-0.011358	
26 H	4.13440000	-2.71330000	-0.00080000	-0.037038	
27 H	5.22560000	1.45440000	0.00090000	-0.039128	

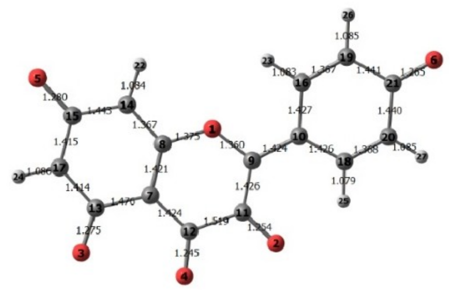

Quercetin radical

xyz -4 2

1 O	-0.25290162	0.63127451	0.00023645	-0.872570	
2 O	0.39503593	-2.92267052	0.08178958	8.762620	
3 O	-4.62210849	-1.41577938	-0.03453427	0.679985	
4 O	-2.23857669	-2.91171490	-0.06550677	1.450964	
5 O	-4.10973583	3.27201262	0.02073644	0.679178	
6 O	4.22579468	2.52488552	-0.01660275	8.734393	
7 O	5.97779856	0.42150984	-0.04235139	16.118501	
8 C	-2.37731839	-0.53259058	-0.01124550	0.269622	
9 C	-1.61249178	0.66482250	0.00380811	0.229279	
10 C	0.47844707	-0.54978105	0.02423763	-2.457229	
11 C	1.89368584	-0.29552889	0.02308437	8.744293	
12 C	-0.17728471	-1.78569537	0.03730748	10.034483	
13 C	-1.69574116	-1.78927164	-0.02038925	0.793493	
14 C	-3.84509448	-0.39699694	-0.01839039	0.484800	
15 C	-2.16188199	1.92756310	0.01425125	1.018526	
16 C	-3.58550740	2.09924847	0.01115180	0.574050	
17 C	2.39396829	1.01943614	0.01046076	-7.186612	2’
18 C	-4.36661633	0.91489601	-0.00257466	-0.571099	
19 C	2.84223619	-1.37308507	0.01969534	7.957986	6’
20 C	3.77093341	1.33383873	-0.00728154	8.104027	
21 C	4.18792055	-1.12574826	-0.00024965	3.682734	5’
22 C	4.73441604	0.19940608	-0.01767541	10.45779	
23 H	-1.51402106	2.79792374	0.02208943	-0.002871	
24 H	1.71110086	1.86090616	0.01237676	-0.024448	
25 H	-5.44934290	1.01898711	-0.00506401	-0.005919	
26 H	2.47534735	-2.38724074	0.02989669	0.013896	
27 H	4.89643674	-1.94979681	-0.00657908	-0.088628	

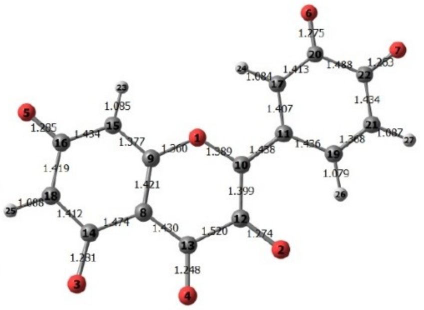

Myricetin radical

xyz -5 2

1 O	0.51460214	0.67601991	0.00032538	-0.949187	
2 O	-0.30837864	-2.84978155	0.10849914	4.763165	
3 O	4.78472086	-1.55851920	-0.00696281	0.388372	
4 O	2.32912513	-2.95192966	0.00170402	1.215553	
5 O	4.47962365	3.14904422	-0.04922365	0.372154	
6 O	-3.80322224	2.85412110	0.09833289	6.245874	
7 O	-5.70260031	0.88282839	-0.05285534	17.323197	
8 C	2.57775576	-0.57979003	0.00102837	0.239710	
9 C	1.86876284	0.64924762	-0.00440518	0.366536	
10 C	-0.27388371	-0.47402376	0.02883309	-4.683581	
11 C	-1.69417288	-0.14377077	0.02403836	9.032743	
12 C	0.31858237	-1.72895438	0.05793033	6.727205	
13 C	1.83183630	-1.80443260	0.01663515	0.806857	
14 C	4.04976925	-0.50715122	-0.01292932	0.320884	
15 C	2.47514364	1.89018481	-0.02026882	0.103212	
16 C	3.90085282	1.99951347	-0.03441194	0.379921	
17 C	-2.08740194	1.21589117	0.06913457	-4.954727	2’
18 C	4.62876626	0.78010797	-0.03047520	-0.378933	
19 C	-2.68661498	-1.14744870	-0.03921931	-5.248749	6’
20 C	-3.43553062	1.62150403	0.05251492	7.356105	
21 C	-4.06353791	-0.84763930	-0.06164335	7.575114	
22 C	-4.47106906	0.57081593	-0.02285043	14.030611	
23 H	1.86394898	2.78701294	-0.02535911	-0.000302	
24 H	-1.34491086	2.00253024	0.12235153	-0.006854	
25 H	5.71532204	0.83448712	-0.03987364	-0.003894	
26 H	-2.40175817	-2.18758961	-0.07333690	0.006098	
27 O	-4.97738942	-1.75187899	-0.11572578	6.520463	

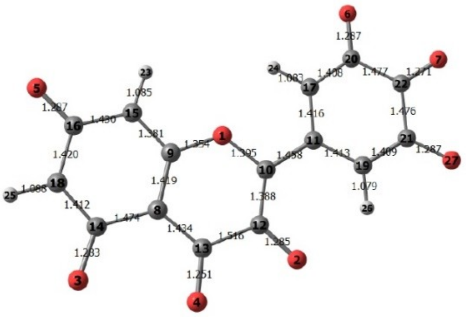

3MQ radical

xyz -3 2

1 O	0.32220375	-0.84701575	-0.15775612	-0.568775	
2 O	4.61043079	1.37121660	0.00683350	0.209471	
3 O	2.15206773	2.78336922	-0.02781744	0.619355	
4 O	4.27996061	-3.33682251	-0.01467130	0.200624	
5 O	-4.13113899	-2.62292451	0.76684089	21.636357	
6 O	-5.90845258	-0.75501415	-0.16767310	19.582886	
7 C	2.41343677	0.39860620	-0.06718094	0.761735	
8 C	1.70047115	-0.82887774	-0.10141029	0.112824	
9 C	-0.41121111	0.29578305	-0.18076868	-2.817553	
10 C	-1.85827591	0.02495195	-0.18599498	10.101030	
11 C	0.20986088	1.49683658	-0.16393742	5.599084	
12 C	1.67919541	1.62919581	-0.08469850	-0.025975	
13 C	3.88084312	0.32057495	-0.01842185	0.088547	
14 C	2.28543004	-2.06856217	-0.08416166	-0.051834	
15 C	3.71707892	-2.18484871	-0.03159192	0.144439	
16 C	-2.31815580	-1.18659412	0.28874442	3.080358	2’
17 C	4.45093245	-0.97165125	-0.00254634	-0.085751	
18 C	-2.79550079	0.98373493	-0.69279795	7.766597	6’
19 C	-3.70898285	-1.52114510	0.32295093	11.976782	
20 C	-4.13493152	0.72100541	-0.69120585	3.040202	5’
21 C	-4.67713668	-0.51118870	-0.18653911	10.404604	
22 H	1.66940913	-2.95970040	-0.11008039	0.000673	
23 H	-1.62833341	-1.93142020	0.66796108	-0.073109	
24 H	5.53522429	-1.03342357	0.03296503	-0.002274	
25 H	-2.42951589	1.91804824	-1.09312371	-0.084448	
26 H	-4.84073653	1.44526511	-1.08468810	-0.104007	
27 O	-0.50760619	2.66955532	-0.18485429	0.462948	
28 C	-0.80049794	3.16324257	1.12551657	0.366748	
29 H	-1.31159568	4.11618496	0.99560567	0.012631	
30 H	-1.45177683	2.46358146	1.65791739	0.015251	
31 H	0.12226688	3.31616841	1.69048746	-0.003128	

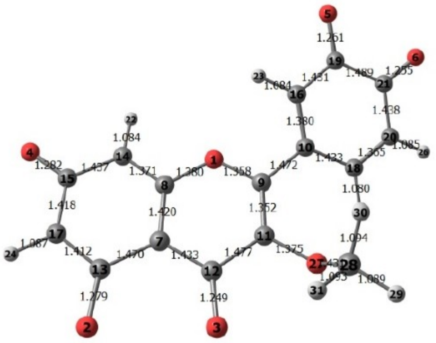

3’MQ radical

xyz -3 2

1 O	0.38545631	0.44495771	0.02726547	2.581666	
2 O	0.09029761	-3.11578281	0.07234064	22.520946	
3 O	4.93190069	-1.16613336	-0.04670765	1.500252	
4 O	2.72096913	-2.89194914	0.07055069	0.791958	
5 O	3.96003593	3.44921007	-0.05011288	1.420264	
6 O	-5.77068721	-0.27209773	-0.10228169	11.238872	
7 C	2.62536182	-0.50696892	0.00339054	0.221857	
8 C	1.74783753	0.61050241	0.00632044	-1.210370	
9 C	-0.22586779	-0.77574740	0.02596860	16.837225	
10 C	-1.64462807	-0.68954232	0.00680732	1.582959	
11 C	0.57014765	-1.95702093	0.04870599	12.610601	
12 C	2.08393836	-1.82237486	0.04260045	0.299016	
13 C	4.07113195	-0.22527898	-0.03056212	0.856801	
14 C	2.16078347	1.91471237	-0.01084388	5.222588	8
15 C	3.56864337	2.23059171	-0.03623386	0.859456	
16 C	-2.26776162	0.60022539	0.03295692	0.828877	2’
17 C	4.46250928	1.13306014	-0.04665617	-0.627249	
18 C	-2.49745954	-1.82473314	-0.04974021	6.543919	6’
19 C	-3.62654247	0.73540296	0.00433720	2.444339	
20 C	-3.86204933	-1.68618127	-0.08417584	1.119052	5’
21 C	-4.51799999	-0.41473216	-0.06361562	7.798469	
22 H	1.43080007	2.71563963	-0.00627401	-0.032391	
23 H	-1.63930869	1.47758155	0.07804502	-0.037334	
24 H	5.52853031	1.34183225	-0.06942824	-0.011184	
25 H	-2.06064921	-2.81068370	-0.07362609	-0.010395	
26 H	-4.50042992	-2.56217676	-0.13207091	-0.049935	
27 O	-4.28886563	1.93086054	0.03480875	0.646337	
28 C	-3.49375263	3.10495137	0.11138527	-0.039585	
29 H	-4.18978801	3.94086546	0.13130633	-0.002852	
30 H	-2.88811554	3.10992196	1.02220702	0.026649	
31 H	-2.83964534	3.19329922	-0.76093610	0.026604	

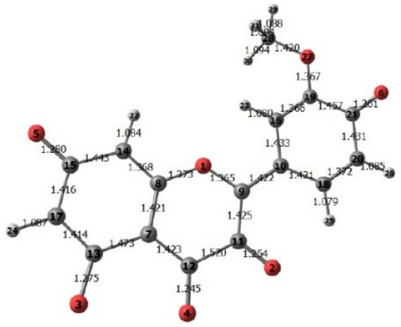

4’MQ radical

xyz -3 2

1 O	0.63941345	0.62691027	0.05050478	5.310653	
2 O	-0.04155927	-2.84206301	-0.25721245	18.793471	
3 O	4.99602422	-1.45863272	-0.00225704	1.348656	
4 O	2.59459497	-2.94338624	-0.03873898	-0.461102	
5 O	4.51231079	3.23066349	0.10557669	1.177521	
6 O	-3.80849189	2.60364790	-0.30610714	-1.243983	
7 C	2.76950387	-0.56193870	0.00850529	0.087209	
8 C	2.01867460	0.64441151	0.03781160	-2.169487	
9 C	-0.09102459	-0.49916068	-0.02083338	22.292134	
10 C	-1.51882227	-0.25561274	-0.01656457	-3.563130	
11 C	0.56814585	-1.76392550	-0.11453278	6.051354	
12 C	2.08885576	-1.80752987	-0.03631325	-0.384578	
13 C	4.23952196	-0.43502828	0.02183731	0.645670	
14 C	2.56373988	1.89465672	0.07163817	6.618285	8
15 C	4.00182373	2.06038180	0.07910749	0.490224	
16 C	-1.99431785	1.06705673	-0.17002833	2.822428	2’
17 C	4.77248391	0.87555319	0.05696678	-0.047020	
18 C	-2.44471739	-1.29396632	0.17429998	9.977154	6’
19 C	-3.35831909	1.40862104	-0.16101882	-2.050481	
20 C	-3.80964897	-1.00278059	0.19480660	-3.415118	5’
21 C	-4.26383752	0.29563633	0.02727457	8.450935	
22 H	1.92224284	2.76856570	0.09576613	-0.048824	
23 H	-1.29377411	1.88076076	-0.31578976	-0.042962	
24 H	5.85535145	0.97036187	0.06256146	-0.010047	
25 H	-2.11497305	-2.30970112	0.31890044	-0.049950	
26 H	-4.51140101	-1.81366807	0.34776213	0.016309	
27 O	-5.58016795	0.65063419	0.02448629	2.257089	
28 C	-6.53681771	-0.38266630	0.20340382	-0.012160	
29 H	-7.51160538	0.09766566	0.15618179	-0.010329	
30 H	-6.45992783	-1.13149865	-0.58943542	0.073391	
31 H	-6.41437233	-0.86692708	1.17587321	0.078346	

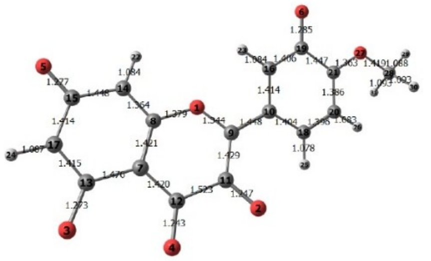

Figure S1. The optimized structure of the test compounds (fully deprotonated radical). The xyz coordinate of the atoms of each molecule (from the second to the fourth column), the spin population in percentage (numbers in red, the fifth column) and the bond lengths given in the depicted molecule (Å). Note that the number here is not the same as that of manuscript, the number in blue (the sixth line) represents the number of carbon used in manuscript and depicted in Figure 1.

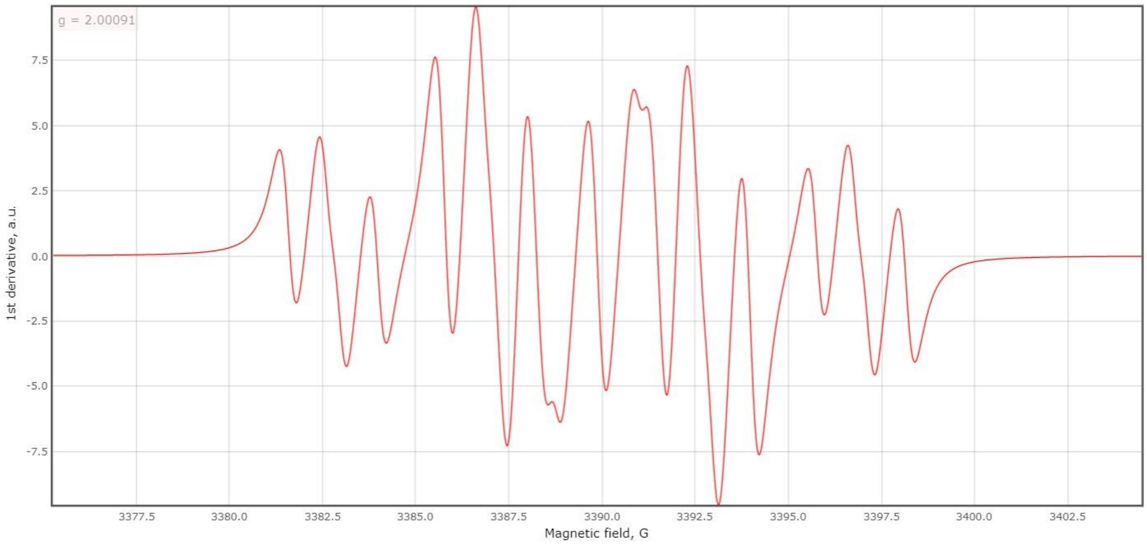

Figure S2. Simulated ESR spectrum of the kaempferol radical using our calculated spin densities. The simulated ESR spectrum is similar as the experimental ESR spectrum of the kaempferol radical Pirker et al. [1]. reported. The simulation was made using eprsimulator (http://www.eprsimulator.org/isotropic.html) with the splitting constants of 4.3, 1.3, 1.0, 4.1, and 5.8, respectively.

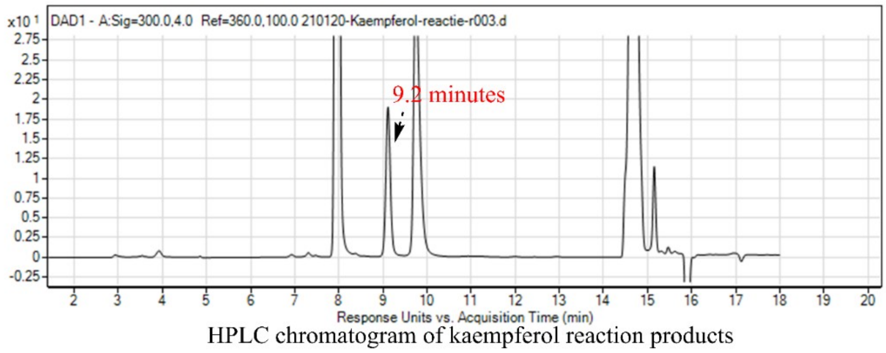

HPLC chromatogram of kaempferol reaction products

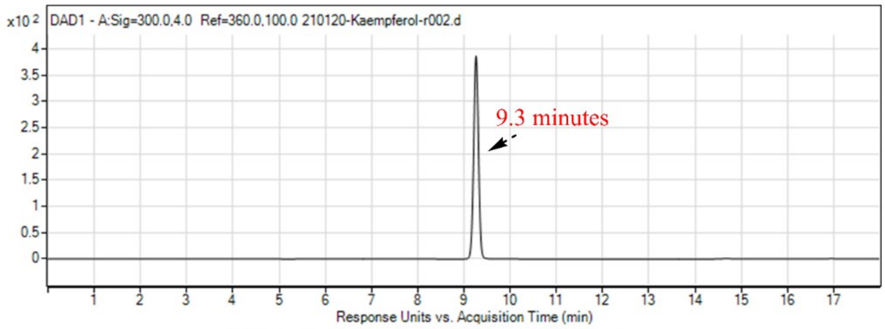

HPLC chromatography of Standard Hydroquinone

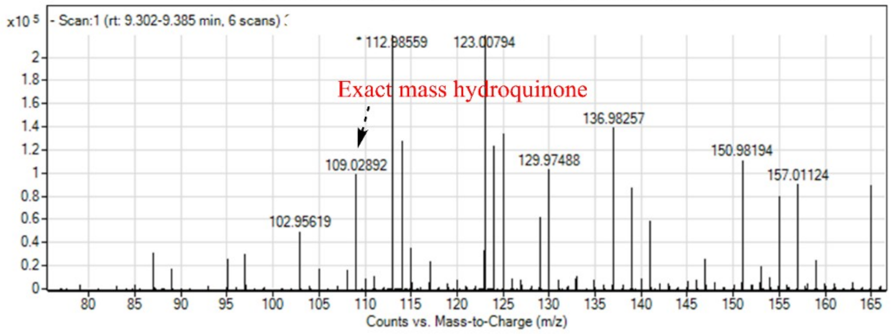

MS of kaempferol reaction products between RT 9.302 and 9.385 minutes

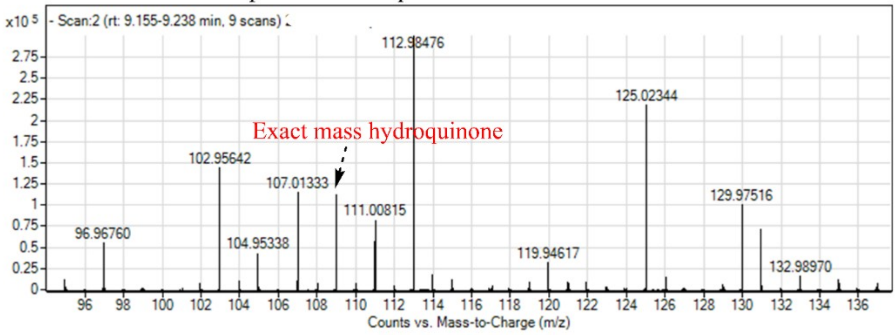

MS of Standard hydroquinone between RT 9.155 and 9.238 minutes

Figure S3. HPLC/MS analysis of a solution of kaempferol incubated for 5 min at high pH, similar to the incubation of kaempferol in the ESR experiment as described in the material and method section of our manuscript. Panel A is the HPLC chromatogram of the Kaempferol solution after the 5 min incubation. In panel B is the HPLC chromatogram of a freshly prepared 2.5 mM hydroquinone solution in a 0.1% formic acid (85%) acetonitril (15%) mixture. The exact mass of the peak at 9.2 min in the kaempferol chromatogram had the same exact mass as the peak of the hydroquinone standard, confirming the formation of hydroquinone out of kaempferol during the incubation. The small difference in retention time incubation between the hydroquinone peak in the chromatogram of the kaempferol incubation and the chromatogram of the hydroquinone reference is probably due to the difference in the composition of solvent of the kaempferol incubation and that of the hydroquinone standard. The formation of hydroquinone in the kaempferol incubation, substantiates the explanation that the ESR signal with kaempferol was derived from the hydroquinone radical.

The conditions of the HPLC analysis are:

The column used for the separation was an Apollo C18 (150 × 4.6 mm, 5 μm) from Grace. The mobile phase was 0.1% formic acid (solvent A) and acetonitrile (solvent B). The linear gradient elution conditions were: start with 95% solvent A ; from 95% to 85% of solvent A in 2 min; from 85% to 78% of solvent A in 7 min; from 78% to 50% of solvent A in 1 min; from 50% to 10% of solvent A in 2 min and this condition was held for 6 min; and from 10% to 95% of solvent A in 1 min which was maintained for 1 min. The oven temperature was set at 25 °C and the injection volume was 5 μL. The flow rate was 0.4 mL/min.

The detection was performed with an Agilent DAD detector (set a 300 nm) on an Agilent 6550 iFunnel Accurate-Mass QuadrupoleTime-of-Flight Mass Spectrometer (Q-TOF MS) through an electrospray interface with Jet Stream technology after separation on a 1290 Infinity UHPLC system (Agilent, Santa Clara, CA, USA). All samples and standards were analysed in negative mode with gas temperature 150 °C, gas flow 20 L/min, nebulizer 25 psig, sheath gas temperature 350 °C, sheath gas flow 12 L/min, Vcap 3000 V and nozzle voltage 1000 V.

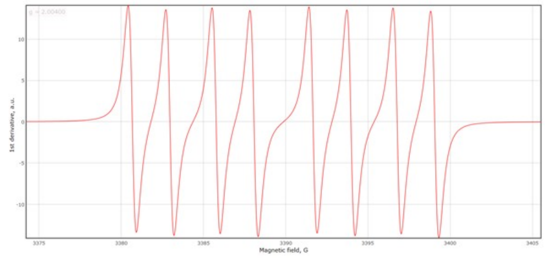

quercetin

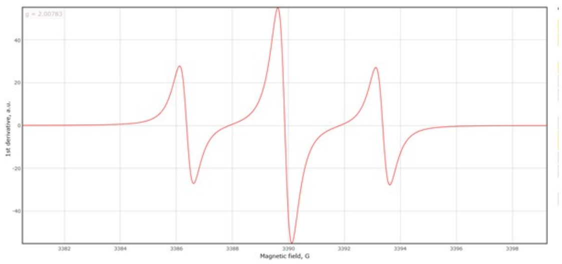

mycricetin

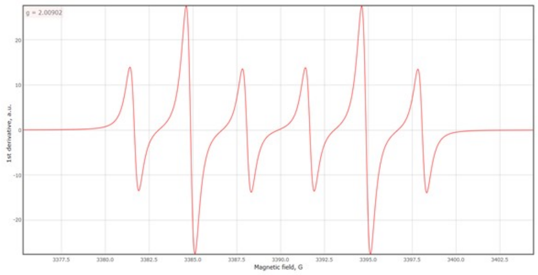

3MQ

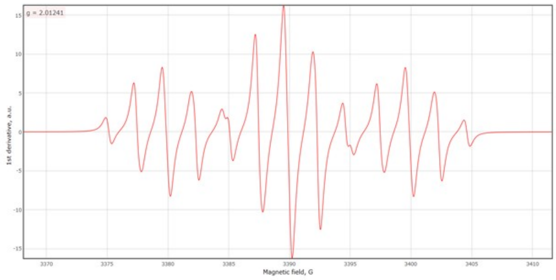

3'MQ

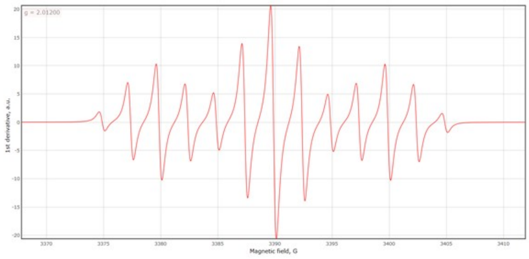

4'MQ

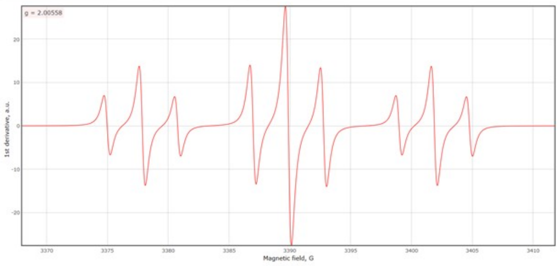

catechol

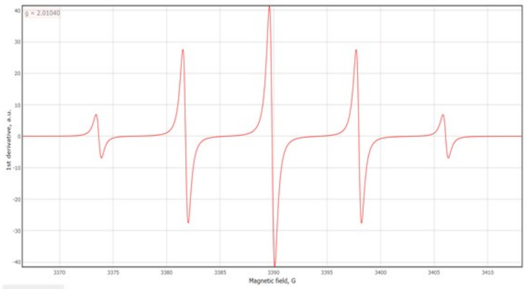

hydroquinone

Figure S4. The simulated ESR spectrum of tested compounds (expect kaemferol) made using eprsimulator (http://www.eprsimulator.org/isotropic.html) with the experimental splitting constants.

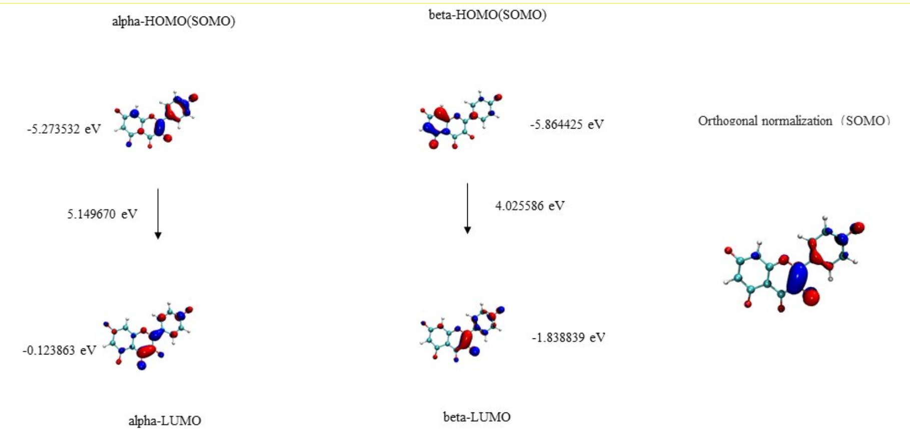

Kaempferol

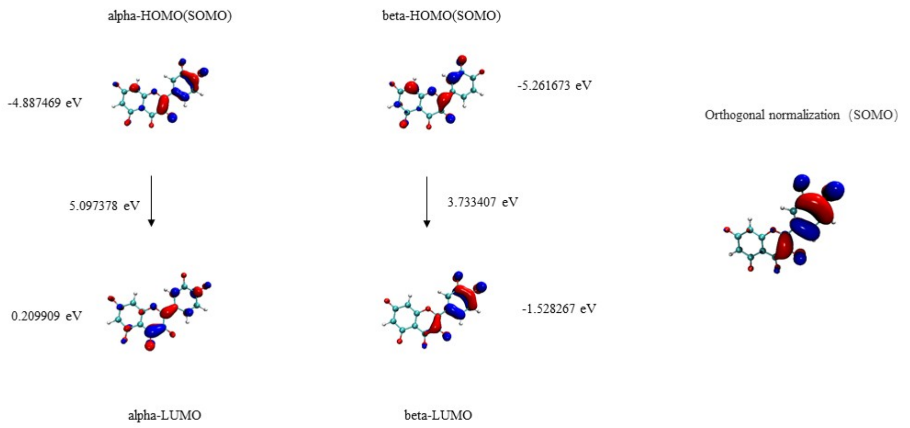

quercetin

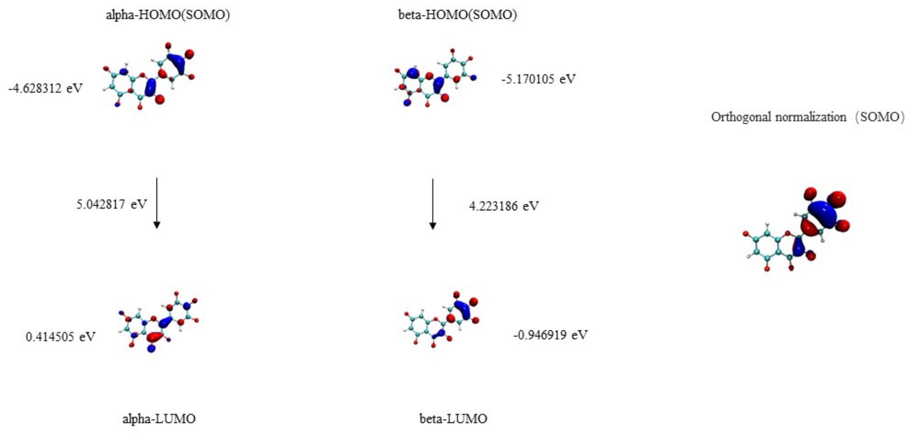

myricetin

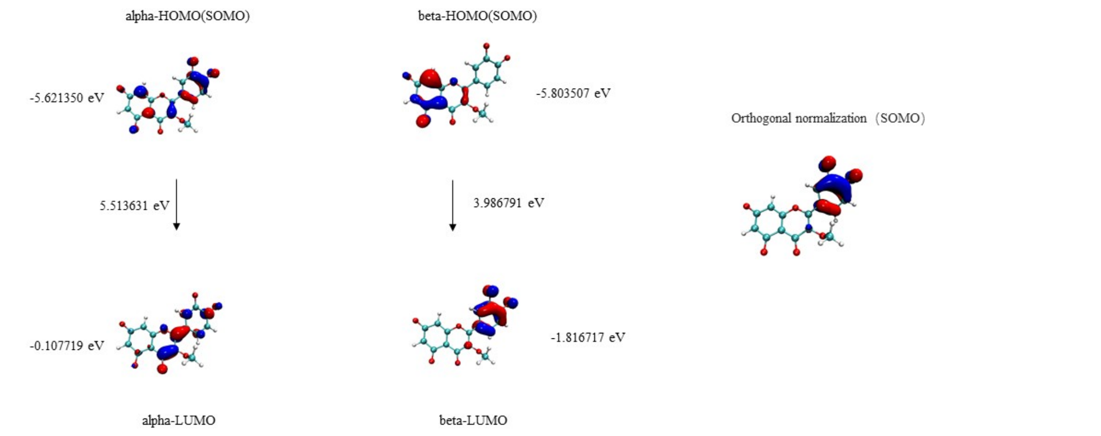
alpha-HOMO(SOMO)
beta-HOMO(SOMO)
-5.621350 eV
-5.803507 eV
Orthogonal normalization（SOMO)
5.513631 eV
3.986791 eV
-0.107719 eV
-1.816717 eV
alpha-LUMO
beta-LUMO

3MQ

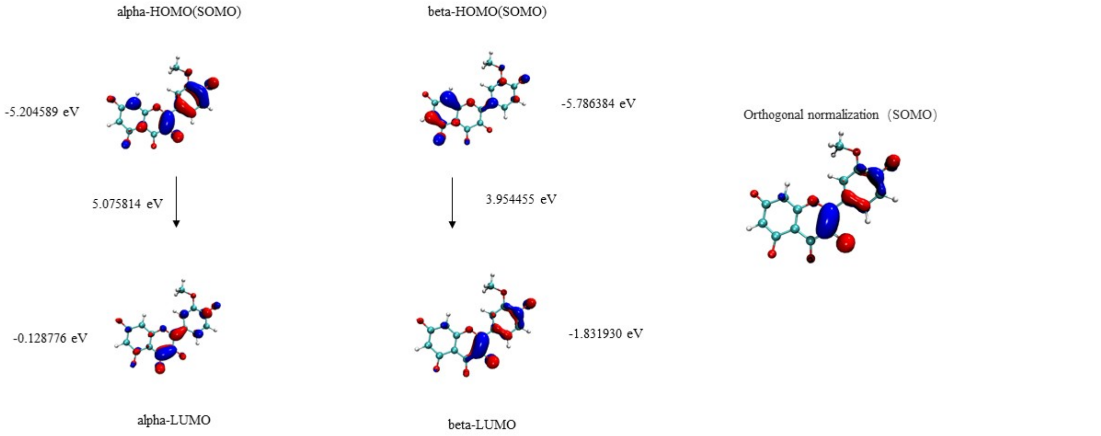
alpha-HOMO(SOMO)
beta-HOMO(SOMO)
-5.204589 eV
-5.786384 eV
Orthogonal normalization（SOMO)
5.075814 eV
3.954455 eV
-0.128776 eV
-1.831930 eV
alpha-LUMO
beta-LUMO

3’MQ

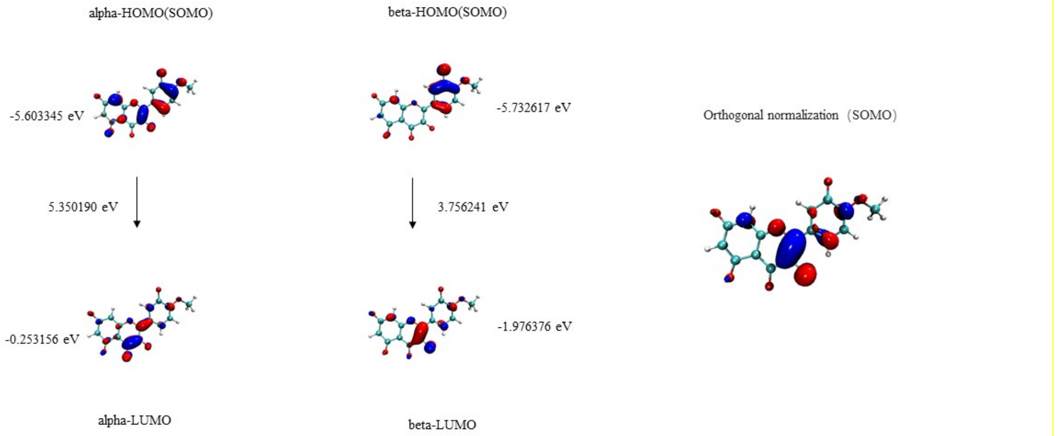
alpha-HOMO(SOMO)
beta-HOMO(SOMO)
-5.603345 eV
-5.732617 eV
Orthogonal normalization（SOMO)
5.350190 eV
3.756241 eV
-0.253156 eV
-1.976376 eV
alpha-LUMO
beta-LUMO

4’MQ

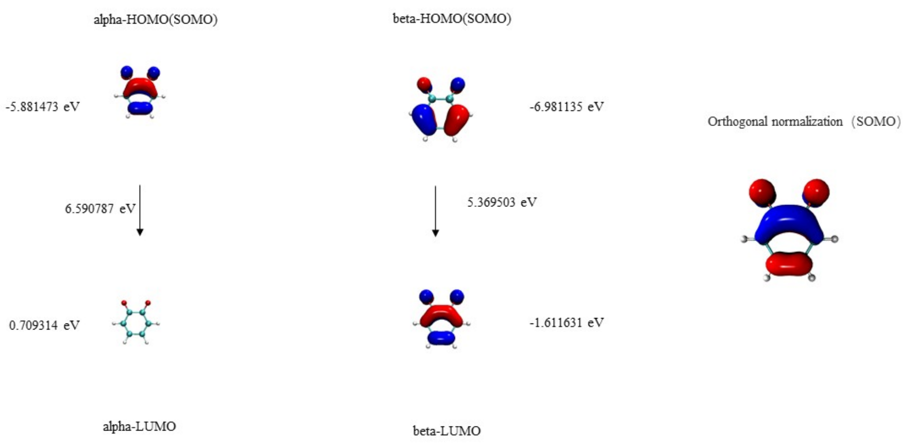

catechol

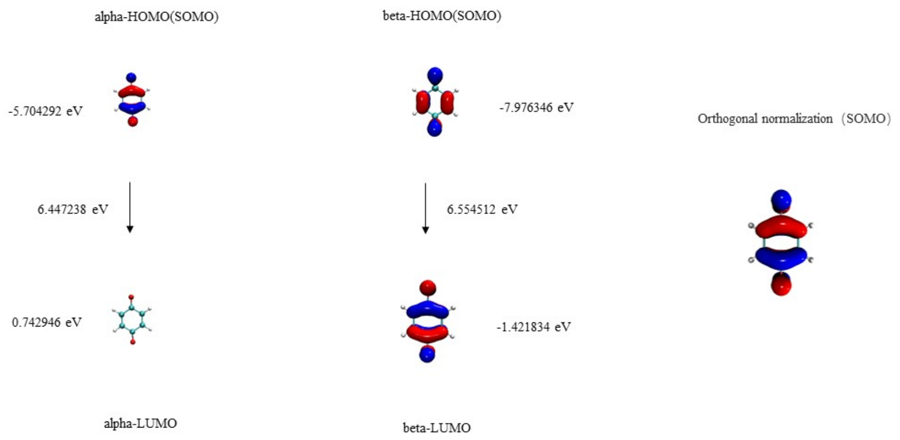

Hydroquinone

Figure S5. The HOMO (SOMO) and LUMO gap of the tested compounds (in the radical form). The gap between alpha-HOMO (SOMO) and alpha-LUMO of kaempferol radical, quercetin radical and myricetin radical are 5.15, 5.10, 5.04 eV, respectively, which indicates that the kaempferol radical is more stable than the quercetin radical and the myricetin radical. The higher alpha energy gap of 4′MQ radical compared with 3′MQ radical indicates the importance of hydroxyl group in Q at the 4′ position. The default isosurface is 0.05 that's why the alpha-LUMO of catechol radical and hydroquinone radical can't be shown. The SOMO maps of tested compounds after orthogonal normalization are shown on the right.

Reference

1. Pirker, K.F.; Stolze, K.; Pirker, K.F.; Stolze, K.; Reichenauer, T.G.; Pirker, K.F.; Stolze, K.; Reichenauer, T.G.; Nohl, H.; Pirker, K.F. Are the biological properties of kaempferol determined by its oxidation products? *Free Radic. Res.* **2006**, 40, 513–521.
